# Functional screening of the *Arabidopsis* 2C protein phosphatases family identifies PP2C15 as a negative regulator of plant immunity by targeting BRI1‐associated receptor kinase 1

**DOI:** 10.1111/mpp.13447

**Published:** 2024-04-01

**Authors:** Zhihong Diao, Rongqian Yang, Yizhu Wang, Junmei Cui, Junhao Li, Qiqi Wu, Yaxin Zhang, Xiaosong Yu, Benqiang Gong, Yan Huang, Guozhi Yu, Huipeng Yao, Jinya Guo, Huaiyu Zhang, Jinbo Shen, Andrea A. Gust, Yi Cai

**Affiliations:** ^1^ Department of Biotechnology and Applied Biology, College of Life Sciences Sichuan Agricultural University Ya'an Sichuan China; ^2^ Chengdu Lusyno Biotechnology Co., Ltd. Chengdu China; ^3^ Guangdong Provincial Key Laboratory of Plant Resources, State Key Laboratory of Biocontrol, MOE Key Laboratory of Gene Function and Regulation, School of Life Sciences Sun Yat‐sen University Guangzhou China; ^4^ Zhejiang A&F University State Key Laboratory of Subtropical Silviculture, School of Forestry and Biotechnology Zhejiang A&F University Zhejiang Hangzhou China; ^5^ Department of the Centre for Plant Molecular Biology, Plant Biochemistry Eberhard Karls University of Tübingen Tübingen Germany

**Keywords:** *Arabidopsis*, BAK1, flg22, PP2C15, PP2C5

## Abstract

Genetic engineering using negative regulators of plant immunity has the potential to provide a huge impetus in agricultural biotechnology to achieve a higher degree of disease resistance without reducing yield. Type 2C protein phosphatases (PP2Cs) represent the largest group of protein phosphatases in plants, with a high potential for negative regulatory functions by blocking the transmission of defence signals through dephosphorylation. Here, we established a PP2C functional protoplast screen using *pFRK1*::*luciferase* as a reporter and found that 14 of 56 PP2Cs significantly inhibited the immune response induced by flg22. To verify the reliability of the system, a previously reported MAPK3/4/6‐interacting protein phosphatase, PP2C5, was used; it was confirmed to be a negative regulator of PAMP‐triggered immunity (PTI). We further identified PP2C15 as an interacting partner of BRI1‐associated receptor kinase 1 (BAK1), which is the most well‐known co‐receptor of plasma membrane‐localized pattern recognition receptors (PRRs), and a central component of PTI. PP2C15 dephosphorylates BAK1 and negatively regulates BAK1‐mediated PTI responses such as MAPK3/4/6 activation, defence gene expression, reactive oxygen species bursts, stomatal immunity, callose deposition, and pathogen resistance. Although plant growth and 1000‐seed weight of *pp2c15* mutants were reduced compared to those of wild‐type plants, *pp2c5* mutants did not show any adverse effects. Thus, our findings strengthen the understanding of the mechanism by which PP2C family members negatively regulate plant immunity at multiple levels and indicate a possible approach to enhance plant resistance by eliminating specific PP2Cs without affecting plant growth and yield.

## Graphical abstract

PP2C15 negatively regulates typical PAMP‐triggered immunity responses such as MAPK3/4/6 activation, defence gene expression, reactive oxygen species bursts, stomatal immunity, callose deposition, and pathogen resistance by dephosphorylating BRI1‐associated receptor kinase 1.

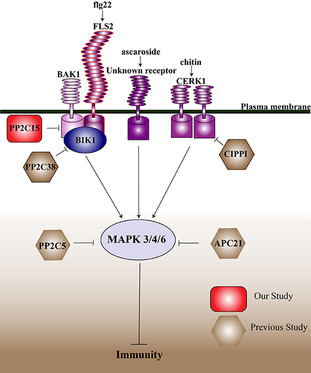

## 1 INTRODUCTION

The recognition of pathogen‐associated molecular patterns (PAMPs) by pattern recognition receptors (PRRs) initiates a complex signalling cascade, resulting in PAMP‐triggered immunity (PTI) (Dangl et al., 2013; Ronald & Beutler, 2010). In plants, PRRs comprise receptor‐like proteins (RLPs) or receptor‐like kinases (RLKs) located at the plasma membrane (PM) (Macho et al., 2014). PRRs usually form dynamic complexes with other RLKs to regulate plant immune signalling (Böhm et al., 2014; Macho & Zipfel, 2014). FLAGELLIN‐SENSITIVE 2 (FLS2) and Pep receptors (PEPRs) are RLKs in *Arabidopsis* with extracellular leucine‐rich repeats (LRRs) that recognize the PAMP flg22, a small peptide conserved at the N‐terminus of bacterial flagellin, and endogenous Peps as damage‐associated molecular patterns (DAMP), respectively (Monaghan & Zipfel, 2012; Zipfel et al., 2004).

The FLS2 and PEPRs are widely used models for studying PTI (Chinchilla et al., 2006; Krol et al., 2010; Robatzek et al., 2006; Yamaguchi et al., 2010). In the resting state, FLS2 and BIK1 interact with related AVRPPHB SUSCEPTIBLE1‐like (PBL) proteins, and BIK1 can also associate with BAK1 (Lu et al., 2011; Macho & Zipfel, 2014; Zhang et al., 2010). When FLS2 senses flg22, BAK1 is recruited to the FLS2/BIK1 complex. BIK1 is subsequently phosphorylated and released from the complex (Lu et al., 2011; Zhang et al., 2010), leading to multiple phosphorylation events. For example, the MAPK and calcium‐dependent protein kinase (CDPK) pathways are triggered, further inducing the expression of defence genes. Increasing evidence suggests that the first substrate downstream of the activated RLK complex at the PM is a receptor‐like cytoplasmic kinase (RLCKs), particularly in the PTI signal transduction pathway (Liu et al., 2013; Lu et al., 2011; Zhang et al., 2010). Furthermore, induced defence responses include the influx of calcium ions and production of reactive oxygen species (ROS) mediated by the NADPH oxidase RBOHD (Kadota et al., 2014; Laluk et al., 2011; Li et al., 2014; Monaghan et al., 2015; Ranf et al., 2014; Zhang et al., 2010), ultimately resulting in plant resistance to pathogenic microorganisms.

BAK1 regulation before and after PAMP sensing has been extensively studied. In the absence of the corresponding PAMPs, BAK1‐INTERACTING RECEPTOR‐LIKE KINASE2 (BIR2) and BAK1‐INTERACTING RECEPTOR‐LIKE KINASE3 (BIR3) prevent BAK1 from forming a complex with PRR, thus negatively regulating plant immunity (Halter et al., 2014; Imkampe et al., 2017). Additionally, the protein phosphatase family 2A (PP2A) holoenzyme can regulate the phosphorylation of BAK1. Overexpression of PP2A‐C4 significantly reduces BAK1 kinase activity and weakens PTI signalling (Segonzac et al., 2014). After ligand binding, the *Arabidopsis* E3 ubiquitin ligases PUB12 and PUB13 are phosphorylated by BAK1 and target FLS2 for degradation via ubiquitination to attenuate the constitutive activation of the immune system (Lu et al., 2011; Smith et al., 2014).

The protein phosphatase 2C (PP2C) family is evolutionarily conserved and includes most plant protein phosphatases (Cao et al., 2016; Singh et al., 2016). PP2C family members are monomeric enzymes that were found to have regulatory functions in abiotic stresses (for example, drought and high salt) in earlier studies, indicating that this protein family plays an important role in plant adaptation to adverse environmental factors (Cui et al., 2023; Fu et al., 2023; Guo, Lu, et al., 2023; Liu et al., 2023; Wang, Li, et al., 2023; Zhang et al., 2023). Most class A members of the PP2C family are involved in the abscisic acid (ABA) pathway. By analysing the ability of all RCAR–PP2C combinations to regulate ABA signalling through transient expression in *Arabidopsis* protoplasts, it was found that the blocking rate of the ABA response by all class A PP2Cs exceeded 90% (Tischer et al., 2017). In the class B family, AP2C1 negatively regulates the wound‐induced MAPK signalling pathway (Ayatollahi et al., 2022). Members of the class C protein family are involved in cell development (Song et al., 2020). In recent years, it has been reported that PP2Cs also participate in the PTI pathways. For example, the *Arabidopsis* PP2C38 negatively regulates PTI by dephosphorylating BIK1 (Couto et al., 2016). AP2C1, AP2C1‐targeted MITOGEN‐ACTIVATED PROTEIN KINASE3 (MAPK3), and MAPK6 are important regulators of plant–nematode interactions (Sidonskaya et al., 2016). Furthermore, CERK1 associates with a previously unknown protein phosphatase, CERK1‐interacting protein phosphatase 1 (CIPP1), to dynamically control chitin‐triggered immunity via a phosphorylation/dephosphorylation cycle at a tyrosine residue (Gong et al., 2019). A PP2C in tomato, Pic1, negatively regulates the phosphorylation of Pti1b, an immunoregulatory factor triggered by flagellin (Giska & Martin, 2019). Relatively little is known about the PP2C‐mediated negative regulation of PTI. Whether other PP2C family members play a role in plant disease resistance and the regulatory mechanisms involved requires further investigation.

Here, a protoplast transient expression system using the *pFRK1*::*luciferase* (*pFRK1*::*LUC*) reporter was established for functional screening of the *Arabidopsis* PP2C protein family. This system was verified using a previously reported MAPK3/4/6‐interacting protein phosphatase, PP2C5, which is involved in the negative regulation of the PTI response. We identified PP2C15 as a negative regulator of pathogen resistance by dephosphorylating BAK1. Based on functional studies of individual genes, our findings suggest that PP2C family members negatively regulate plant immunity by targeting diverse signalling components and play different roles in regulating plant growth and seed development.

## 2 RESULTS

### 2.1 Establishment and verification of a PP2C functional protoplast screen

**FIGURE 1:**
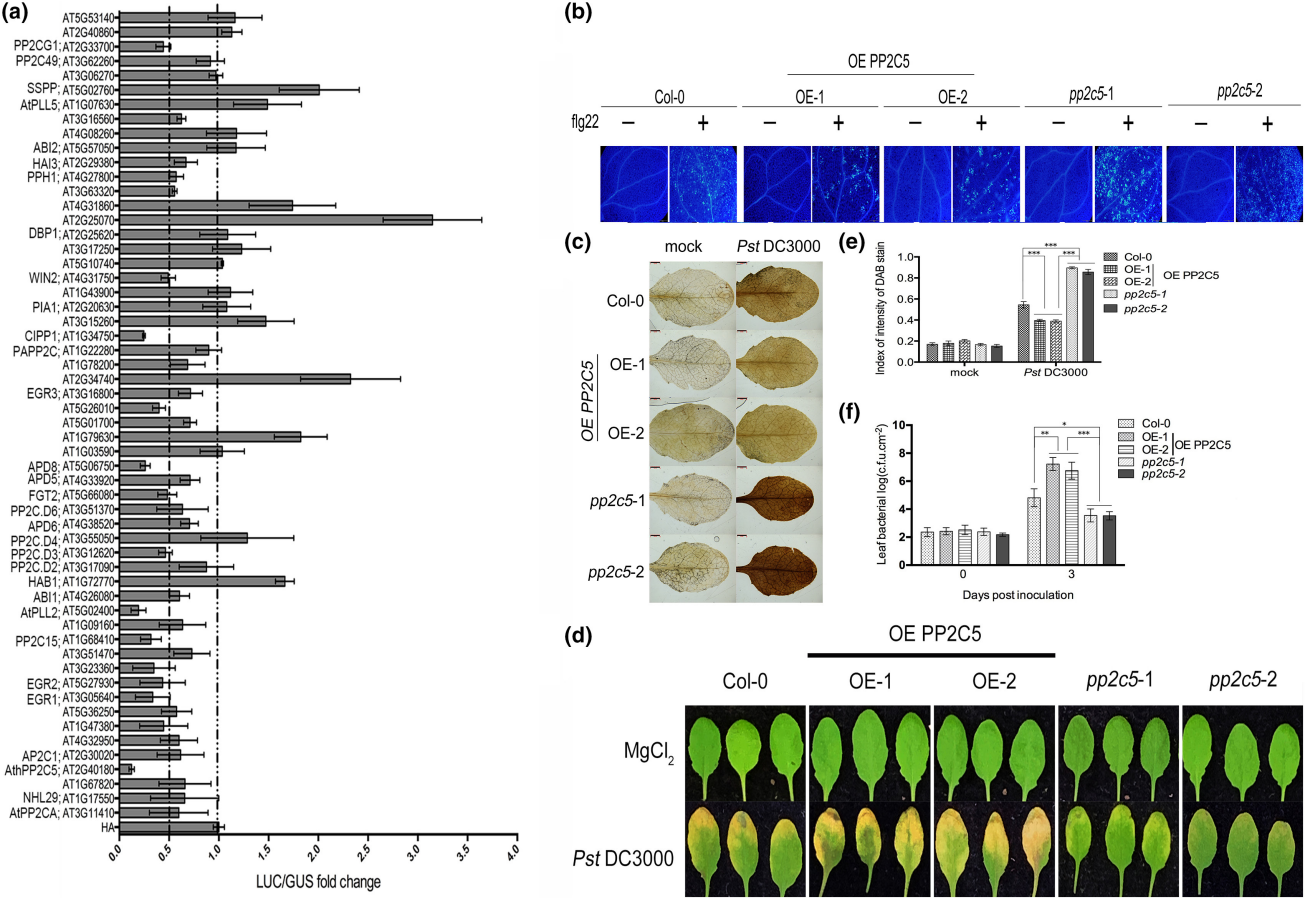
Establishment and verification of PP2C functional protoplast screen by PP2C5. (a) Protoplast screening of 56 PP2C genes in *Arabidopsis* protoplasts, using *pFRK1*::*LUC* as a reporter. *n* = 3. (b) *Arabidopsis* seedlings treated with 100 nM flg22 for 20 h were stained with 0.01% aniline blue. *n* = 3; Bar = 100 μm. (c) Hydrogen peroxide accumulation was detected using 3,3′‐diaminobenzidine (DAB). *n* = 3; Bar = 2 mm. (d) Leaf phenotypes of *Arabidopsis* injected with 10^5^ cfu/mL *Pseudomonas syringae* pv. *tomato* (Pst) DC3000 containing 300 nM flg22. *n* = 3; Bar = 2 mm. (e) Analysis of DAB staining using the ImageJ software. *n* = 3. (f) Analysis of bacterial numbers on Days 0 and 3 after the injection of pathogenic bacteria. *n* = 3. *, ** and *** indicates *p* < 0.05, *p* < 0.01 and *p* < 0.001, respectively (Student's *t* test).

**FIGURE 2:**
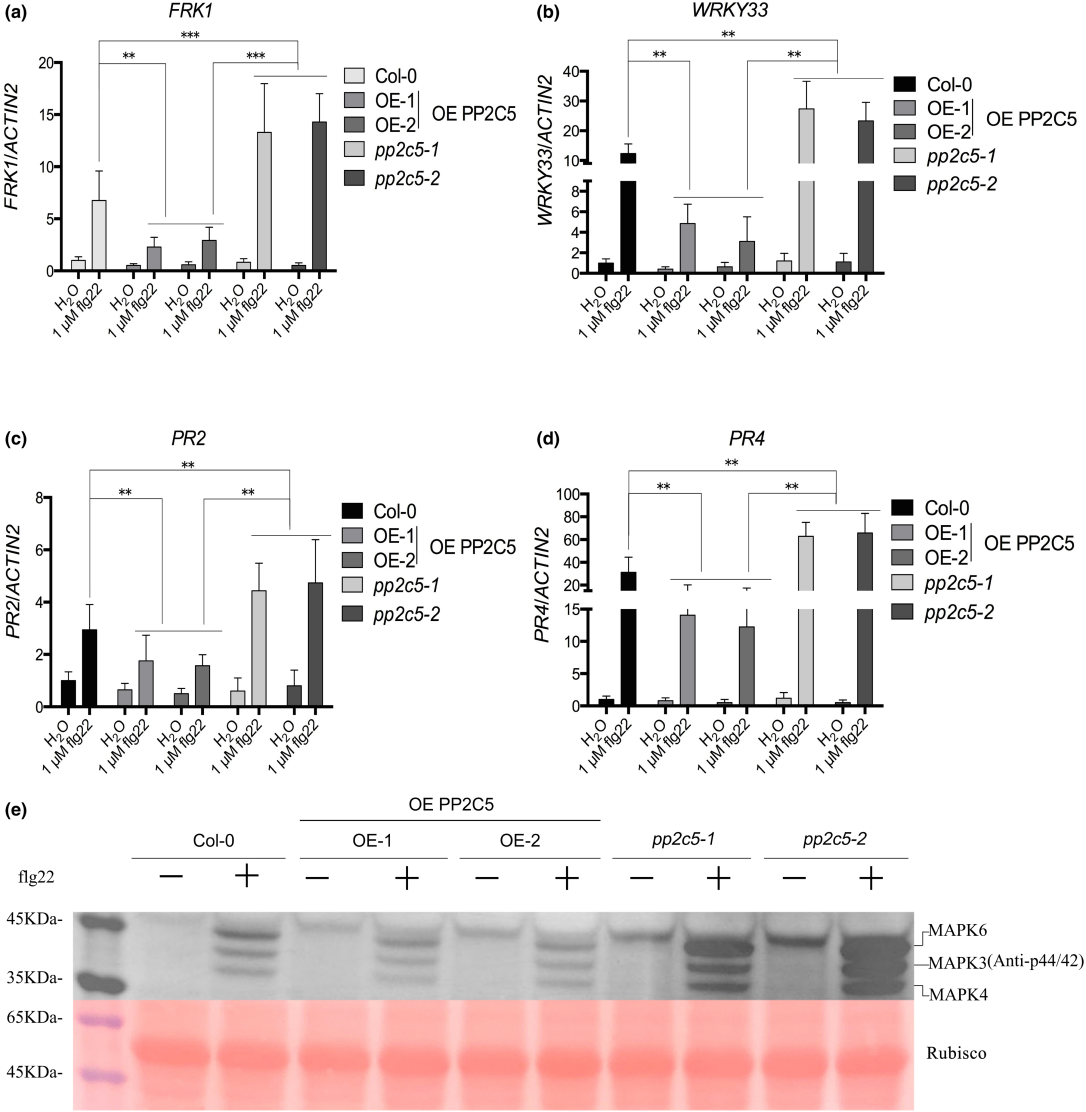
PP2C5 negatively regulates the expression of disease resistance‐related genes and the phosphorylation level of MAPK3/4/6. (a–d) Expression levels of disease resistance‐related reporter genes *FRK1*, *WRKY33*, *PR2*, and *PR4* in PP2C5 overexpression (OE) and mutant plants. *n* = 3. (e) MAPK3/4/6 phosphorylation levels in different plants, detected by western blotting. Rubisco serves as the loading control. *n* = 3; ** and *** indicates *p* < 0.01 and *p* < 0.001, respectively (Student's *t* test).

Most studies of *Arabidopsis* PP2C family members have focused on abiotic stress. However, several PP2C family members have been found to play important roles in plant immunity (Couto et al., 2016; Sidonskaya et al., 2016). We aimed to explore whether other PP2C members play a role in regulating plant immunity, using a protoplast screen with the *pFRK1*::*LUC* system as a reporter. The results showed that among the 56 PP2Cs, 14 strongly inhibited *pFRK1*::*LUC* reporter activity (less than 1/2 of the control activity), and 22 had some, but not significant, inhibitory effects (inhibition between 1/2 and 1; Figure 1a). PP2C5 (AT2G40180), which acts as a MAPK3/4/6 phosphatase and affects the ABA response and PTI‐mediated effector‐triggered immunity (ETI) suppression (Brock et al., 2010; Wang et al., 2023b), showed the strongest inhibitory effect in our screen. Therefore, we studied whether PP2C5 could regulate the PTI response in PP2C5 overexpression plants and mutants, which was verified by reverse transcription‐quantitative PCR (RT‐qPCR) and western blot analysis (Figure S2). In the PP2C5 overexpression lines, flg22 induced a much weaker immune response than that in the wild‐type (Col‐0) plants, including the accumulation of callose and resistance to *Pseudomonas syringae* pv. *tomato* (Pst) DC3000, whereas *pp2c5–1* and *pp2c5–2* mutants showed opposite effects (Figure 1b–f). After treatment with flg22, the expression levels of the disease resistance‐related genes *FRK1*, *WRKY33*, *PR2*, and *PR4* were significantly reduced in PP2C5 overexpression lines, whereas the two *pp2c5* mutants showed significant upregulation of these four genes (Figure 2a–d).

The MAPK cascade is involved in various physiological, developmental, and hormonal responses in plants. Molecular and biochemical studies using MAPK‐specific antibodies showed that when plants are subjected to biological or biotic stresses such as pathogen invasion, trauma, drought, high salt, and high or low permeability, the MAPK pathway is activated (Lin et al., 2021; Romeis, 2001; Segonzac & Zipfel, 2011; Zhang & Klessig, 2001). While overexpression of PP2C5 significantly inhibited the phosphorylation and activation of MAPKs, *pp2c5* mutants showed the opposite effects (Figure 2e). Thus, PP2C5 acts as a negative regulator of plant PTI response.

### 2.2 Overexpression of PP2C15 negatively regulates typical plant immune responses

**FIGURE 3:**
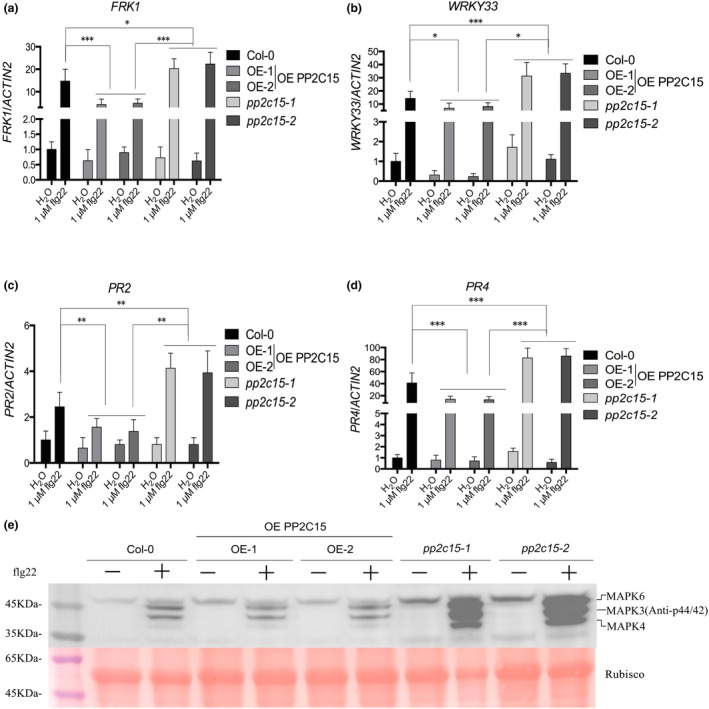
PP2C15 negatively regulates the expression of disease resistance‐related genes and the phosphorylation level of MAPK3/4/6. (a–d) Expression levels of disease resistance‐related reporter genes *FRK1*, *WRKY33*, *PR2*, and *PR4* in PP2C15 overexpression (OE) and mutant plants. *n* = 3. (e) MAPK3/4/6 phosphorylation levels in different plants, detected by western blotting. Rubisco serves as the loading control. *n* = 3; *, ** and *** indicates *p* < 0.05, *p* < 0.01 and *p* < 0.001, respectively (Student's *t* test).

After verifying the protoplast screen, the functions of unreported genes that exhibited strong inhibitory effects during screening were further studied. We identified the top 20 genes co‐expressed with PP2C15 using the *Arabidopsis* RNA‐Seq database (http://ipf.sustech.edu.cn/pub/athrdb/) (Zhang et al., 2020). Gene network and GO enrichment analyses were performed using the STRING database (http://string‐db.org) (Szklarczyk et al., 2021). We found that the regulation of the defence response to bacteria was significantly enriched (Figure S3). Therefore, we inferred that PP2C15 may be involved in the negative regulation of *Arabidopsis* immunity against bacterial infections. We constructed PP2C15 overexpression lines (OE PP2C15) and identified two *pp2c15* mutants. Transgenic lines and mutants were verified using RT‐qPCR and western blot analyses (Figure S4). After treatment with flg22, activation of the MAPK cascade and expression levels of the disease resistance‐related genes *FRK1*, *WRKY33*, *PR2*, and *PR4* were significantly reduced in OE PP2C15, whereas the two *pp2c15* mutants showed significant upregulation of MAPK phosphorylation and expression of the four genes (Figure 3a–e). When transiently expressed in *Nicotiana benthamiana* leaves, PP2C15‐GFP was localized to the cytoplasm and nucleus, similar to GFP (Figure S5).

**FIGURE 4:**
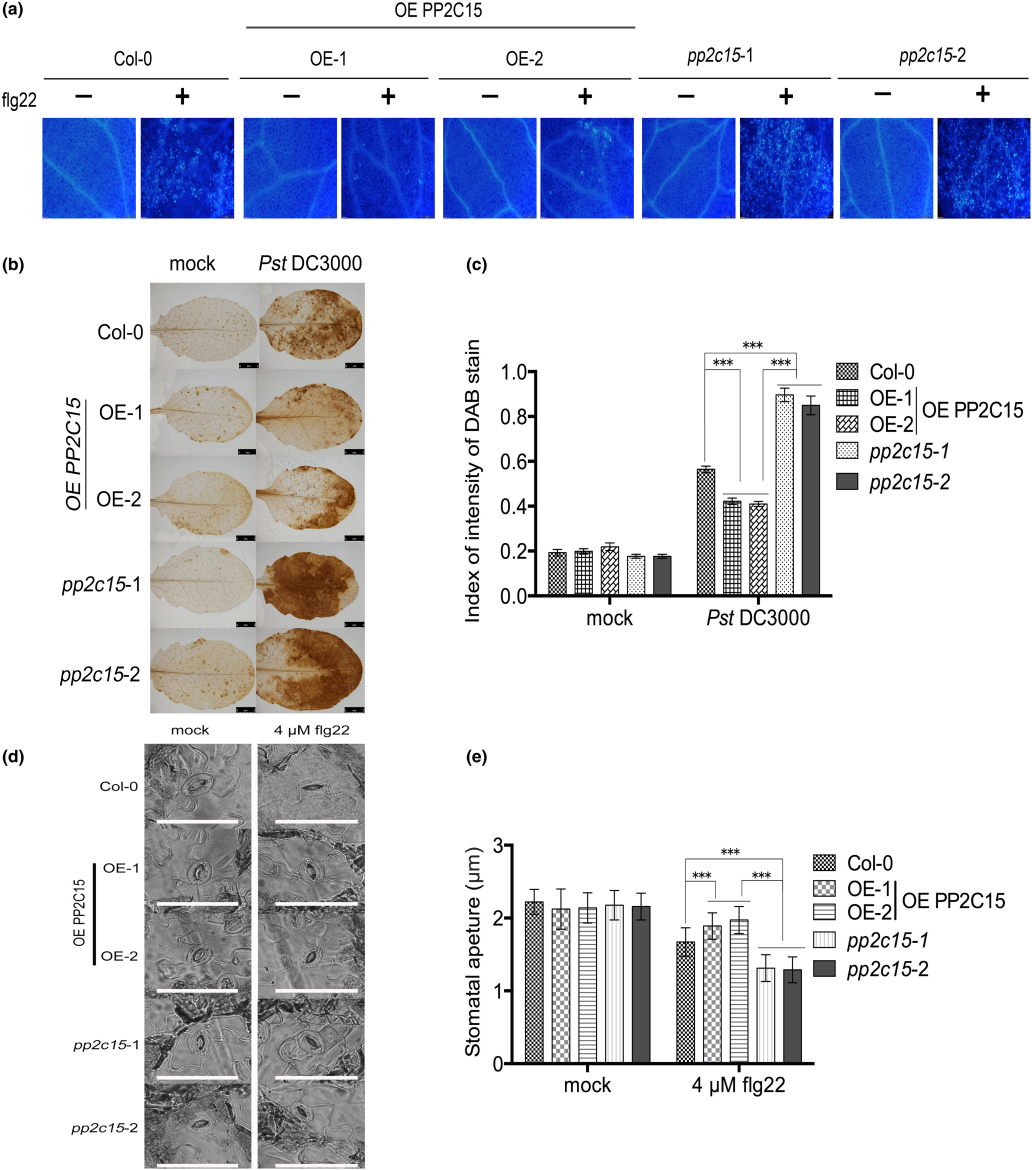
PP2C15 negatively regulates callose accumulation, reactive oxygen species (ROS) burst, and stomatal openness. (a) *Arabidopsis* seedlings treated with 100 nM flg22 for 20 h were stained with 0.01% aniline blue. *n* = 3; bar = 100 μm. (b) Hydrogen peroxide accumulation was detected using 3,3′‐diaminobenzidine (DAB). *n* = 3; bar = 2 mm. (c) Analysis of DAB staining using the ImageJ software. *n* = 3. (d) The stomatal opening of Col‐0, OE PP2C15 (OE‐1, OE‐2), and *pp2c15* in 4‐week‐old *Arabidopsis* leaves treated with 4 μM flg22 were compared with water treatment. *n* = 3; bar = 20 μm. (e) Statistics of stomatal apertures. The results were mean ± standard deviation (*n* > 100); *** indicates *p* < 0.001 (Student's *t* test).

When a PRR senses a PAMP, it activates a downstream immune response, including the accumulation of callose, rapid phosphorylation of RLCK, ROS burst, and the influx of calcium ions across the cell membrane (Tang et al., 2017). Additionally, stomatal closure helps prevent pathogens from entering leaf tissue (Melotto et al., 2008). Callose accumulation in OE PP2C15 was significantly lower than that in wild‐type plants and *pp2c15* mutants (Figure 4a). Simultaneously, when Pst DC3000 was injected into *Arabidopsis* leaves, the overexpression of PP2C15 significantly inhibited ROS production as seen upon 3,3′‐diaminobenzidine (DAB) staining (Figure 4b,c). Similarly, after 4 μM flg22 treatment, stomatal openings in OE PP2C15 were significantly larger than those in Col‐0 and *pp2c15* mutants (Figure 4d,e). Therefore, PP2C15 negatively regulates the typical immune responses.

### 2.3 Overexpression of PP2C15 impacts *Arabidopsis* resistance to bacteria

**FIGURE 5:**
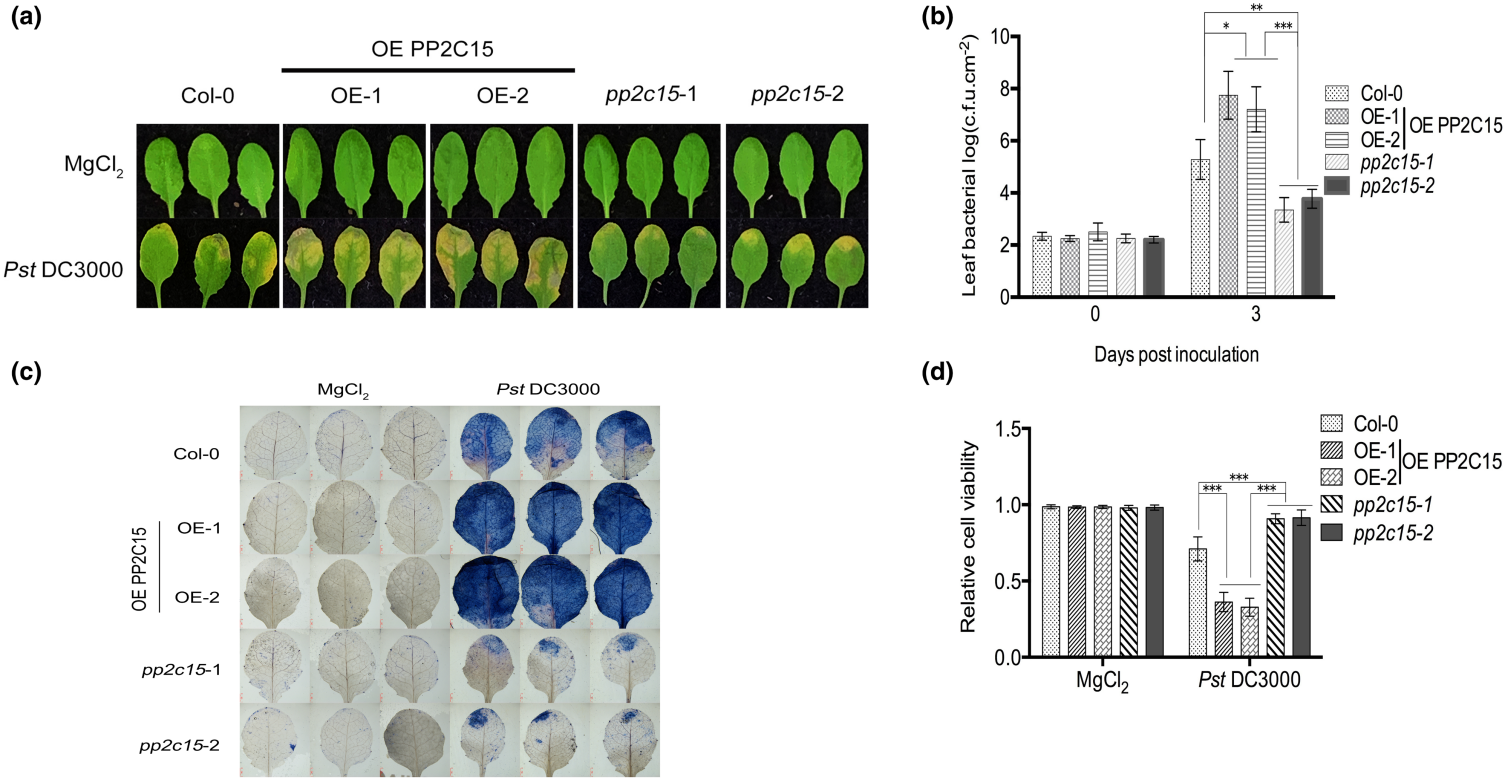
PP2C15 negatively regulates plant immunity. (a) Leaf phenotypes of *Arabidopsis* injected with 10^5^ cfu/mL *Pseudomonas syringae* pv. *tomato* (Pst) DC3000 containing 300 nM flg22. *n* = 3; bar = 2 mm. (b) Analysis of bacterial numbers on Days 0 and 3 after the injection of pathogenic bacteria. *n* = 3. (c) and (d) Evans blue staining and relative cell activity were observed 3 days after the injection of 10^5^ cfu/mL Pst DC3000 containing 300 nM flg22. *n* = 3; bar = 2 mm. *, ** and *** indicates *p* < 0.05, *p* < 0.01 and *p* < 0.001, respectively (Student's *t* test).

Next, we investigated whether PP2C15 depletion or overexpression affected the resistance of *Arabidopsis* to bacterial infection or infection‐related cell death. The results showed that the resistance of two independent OE PP2C15 lines to Pst DC3000 was lower than that of Col‐0 and mutants (Figure 5a,b), and the relative cell death in the overexpression lines was higher (Figure 5c,d). These results suggest that PP2C15 negatively regulates the resistance of *Arabidopsis* to pathogens and that overexpression of this gene accelerates the mass death of plant cells during pathogen infection.

### 2.4 PP2C15 directly interacts with and dephosphorylates BAK1

**FIGURE 6:**
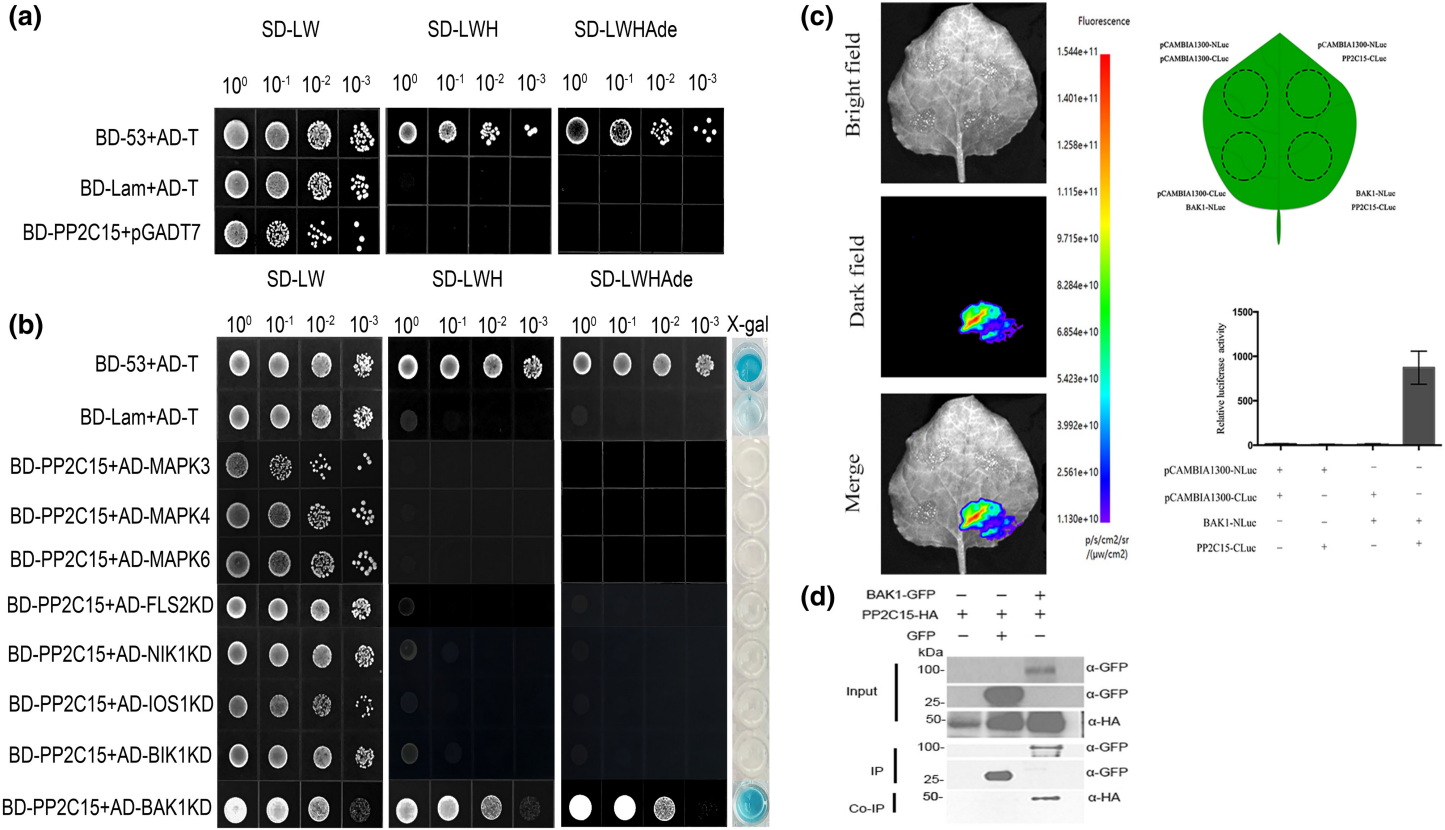
PP2C15 interacts with BAK1 kinase domain (KD) but not MAPK3/4/6, FLS2 KD, NIK1 KD, IOS1 KD, or BIK1 KD. (a) BD‐PP2C15 showed no self‐activation in yeast ywo‐hybrid assay. *n* = 3. (b) PP2C15 interacts with BAK1 KD, but not with MAPK3/4/6, FLS2 KD, NIK1 KD, IOS1 KD, or BIK1 KD. *n* = 3. (c) Luciferase complementation assay verification of PP2C15 interaction with BAK1 KD. *n* = 3. (d) Co‐immunoprecipitation verification of PP2C15 interaction with BAK1 KD. *n* = 3.

Although overexpression of PP2C15 greatly reduced the activation of MAPKs, it is unclear whether PP2C15 acts on MAPKs or their upstream components. Therefore, we first tested whether PP2C15 interacts with MAPK3/4/6 using yeast two‐hybrid (Y2H) assays. PP2C15 neither self‐activated nor interacted with MAPK3/4/6 (Figure 6a,b). Previous studies have identified multiple signalling components that act upstream of the MAPK cascade, including FLAGELLIN SENSING2 (FLS2), BAK1, IMPAIRED OOMYCETE SUSCEPTIBILITY1 (IOS1), and NUCLEAR SHUTTLE PROTEIN‐INTERACTING KINASE 1 (NIK1) (Feng et al., 2012; Li et al., 2019; Liu et al., 2013; Yeh et al., 2016; Zhang et al., 2010; Zou et al., 2018). Therefore, we tested the interactions between PP2C15 and BIK1 and the kinase domains (KDs) of FLS2, BAK1, IOS1, and NIK1. The results showed that PP2C15 specifically interacted with BAK1 KD but not with any other tested proteins (Figure 6b). In order to exclude the possibility that the interaction between BD‐PP2C15 and AD‐BAK1 KD is caused by the self‐activation of AD‐BAK1 KD, we verified that AD‐BAK1 KD did not self‐activate in the Y2H assay (Figure S6). The interaction between PP2C15 and BAK1 was further confirmed by co‐immunoprecipitation (Co‐IP) and luciferase complementation assay (LCA) (Figure 6c,d). BAK1/SERK3 (Somatic Embryogenesis Receptor Kinase 3) belongs to the SOMATIC EMBRYOGENESIS RECEPTOR KINASE (SERK) protein family, which has five members and functions redundantly in plant immunity (Albrecht et al., 2008; Brandt & Hothorn, 2016; Karlova et al., 2006; Liu et al., 2020; Peng & Kaloshian, 2014). Therefore, we verified the interaction between PP2C15 and SERK1/2/4/5. The results showed that PP2C15 only interacted with BAK1 KD but not with any other tested SERK KDs, which indicated a very specific recognition between PP2C15 and its substrate (Figure S7).

**FIGURE 7:**
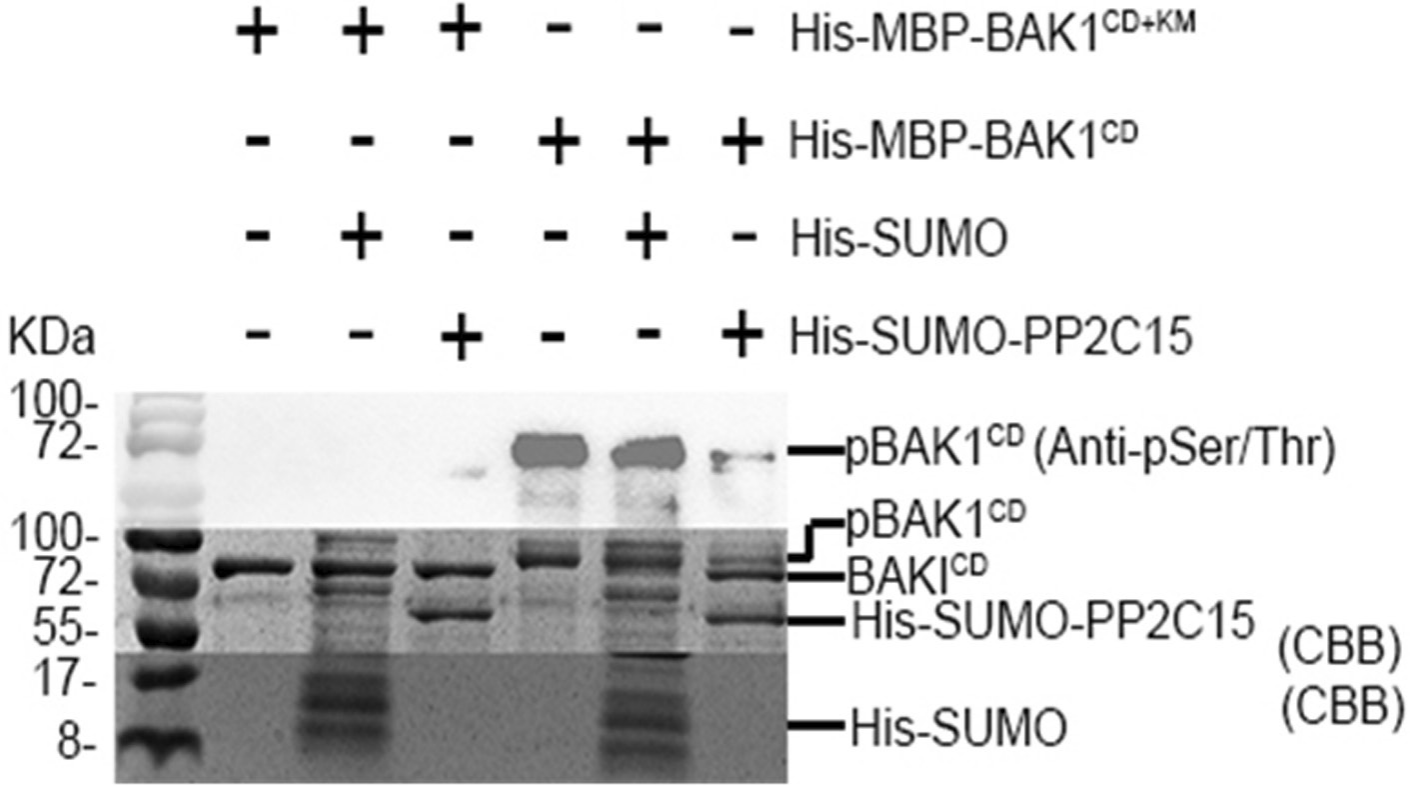
PP2C15 negatively regulates BAK1 cytoplasmic domain (CD) phosphorylation. In vitro phosphatase assay demonstrated that PP2C15 dephosphorylate BAK1 CD. CBB, Coomassie brilliant blue staining. HIS‐SUMO and BAK1 ^CD+Km^ were used as negative controls. pBAK1^CD^: phosphorylated BAK1; BAK1 ^CD+Km^: kinase‐dead (Km) BAK1 CD. *n* = 3.

As a protein phosphatase, PP2C15 may inhibit downstream signalling by dephosphorylating BAK1, thereby achieving negative immunological regulation. To test this hypothesis, we performed an in vitro phosphatase assay using purified recombinant proteins. His‐SUMO‐tagged PP2C15 significantly reduced the phosphorylation level of the BAK1 cytoplasmic domain (CD), as detected by an anti‐pSer/Thr antibody, and caused a gel shift in BAK1^CD^ due to the removal of the phosphate group (Figure 7). We also attempted to analyse the phosphorylation status of BAK1 in vivo; however, we were unable to detect the phosphorylation of BAK1 with or without PP2C15 transient co‐expression in *N. benthamiana* leaves, possibly because of the relatively low activity of BAK1 or the sensitivity of the system (data not shown). Furthermore, we examined the effects of PP2C15 on *pFRK1*::*LUC* expression in Col‐0 and *bak1‐4* protoplasts with and without flg22 and no significant reduction on LUC activity was detected in the absence of flg22, as the BAK1‐mediated PTI signalling cascade was not activated. In addition, flg22 did not effectively induce the expression of *pFRK1*::*LUC* in *bak1‐4* and overexpression of PP2C15 did not cause a significant difference in LUC activity compared with the control vector (Figure S8). Thus, PP2C15 negatively regulates BAK1‐mediated PTI responses via dephosphorylation of BAK1.

### 2.5 PP2C5 and PP2C15 differentially affect growth and seed phenotypes in *Arabidopsis*

**FIGURE 8:**
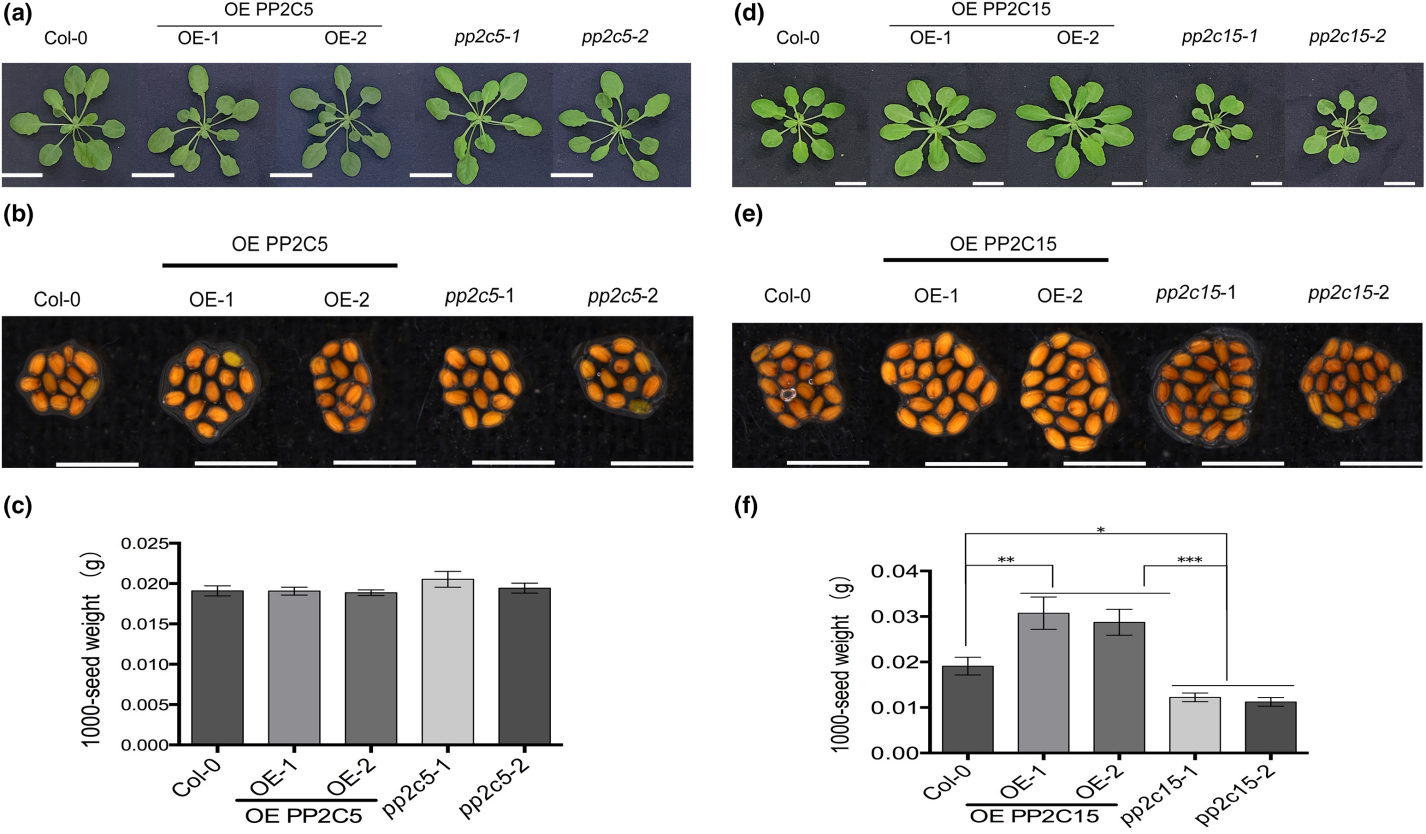
PP2C5 and PP2C15 differentially affect growth and seed phenotypes in *Arabidopsis*. (a) Phenotypes of PP2C5 overexpression (OE) and mutant plants. Bar = 1 cm. (b) and (c) Phenotypes of PP2C5 overexpressing and mutant plant seeds. *n* = 3; bar = 1 mm. (d) Phenotypes of PP2C15 overexpression and mutant plants. Bar = 1 cm. (e) and (f) Phenotypes of PP2C15 overexpression and mutant plant seeds. *n* = 3; bar = 1 mm. *, **, and *** indicates *p* < 0.05, *p* < 0.01, and *p* < 0.001, respectively (Student's *t* test).

To determine whether overexpression or mutation of PP2C5 or PP2C15 affects plant phenotype, we recorded the size of 4‐week‐old *Arabidopsis* rosette leaves and the weight of 1000 mature seeds. As shown in Figure 8, no difference in leaf disc size between OE PP2C5, *pp2c5*, and Col‐0 was observed (Figure 8a); however, *pp2c15* had smaller leaf discs than that of OE PP2C15 and Col‐0 (Figure 8d). While no difference was observed in the 1000‐seed weight between OE PP2C5, *pp2c5*, and Col‐0 (Figure 8b,c), *pp2c15* had a lower 1000‐seed weight than those in OE PP2C15 and Col‐0 (Figure 8e,f).

## 3 DISCUSSION

In mammals, immune signalling must be initiated at a specific time and intensity; otherwise, excessive immune activation leads to autoimmune diseases (Coll & O'Neill, 2010; Kondo et al., 2012). Similarly, plants grow better when there is a fine balance between immunity and growth (Belkhadir et al., 2014; Lozano‐Durán & Zipfel, 2015). An effective plant immune response involves the transmission of immune signals via protein kinases and phosphatases, which regulate the function of target proteins through phosphorylation and dephosphorylation. In addition to protein kinases, an increasing number of studies have revealed that protein phosphatases are also important in the regulation of dynamic signal transduction networks in plants (Ayatollahi et al., 2022; Couto et al., 2016; Macho et al., 2014; Segonzac et al., 2014; Singh et al., 2016; Tischer et al., 2017).

### 3.1 PP2C protein family members negatively regulate PTI at multiple targets

In plants, protein serine/threonine phosphatases, especially PP2Cs, play key roles in the protein phosphatase family (Umbrasaite et al., 2011). Earlier studies on PP2Cs focused on abiotic stress, whereas relatively few studies had been conducted on biotic stress. Only a few PP2C family members had been identified to be involved in the PTI pathway (Couto et al., 2016; Giska & Martin, 2019; Wang, Wei, et al., 2023). However, considering their abundance and diverse functions, it is likely that some important functional PP2Cs involved in the regulation of PTI signalling pathways had not yet been identified. Therefore, we systematically screened *Arabidopsis* PP2Cs using rapid protoplast screening technology. This approach has been widely used to study the functional components of plant immunity (Kovtun et al., 2000; Sheen, 2001; Yoo et al., 2007). In this study, we screened 56 PP2Cs in the protoplast system using *pFRK1*::*LUC* as a reporter and identified 14 *PP2C* genes that significantly inhibited the expression of *pFRK1*::*LUC* (Figure 1a). Among these 14 genes, PP2C38 was reported to negatively regulate PTI by targeting BIK1 (Couto et al., 2016). PP2C5, a previously identified MAPK3/4/6 phosphatase (Brock et al., 2010), exerted the most significant inhibitory effect. From the functional analysis of overexpression lines and mutants of PP2C5, we confirmed its negative regulatory role in plant immune responses (Figure 1b–f).

Through protoplast screening and bioinformatics analysis, we found that a newly identified PP2C, PP2C15, significantly suppressed *pFRK1*::*LUC* reporter expression and was transcriptionally upregulated upon bacterial infection (Figure 1a; Figure S3). Additionally, PP2C15 co‐expressed genes were highly coordinated with plant defence responses (Figure S3). Further functional analysis of PP2C15 confirmed its negative regulatory role in PTI pathways such as MAPK activation, defence gene expression, ROS burst, stomatal immunity, callose deposition, and pathogen resistance. To reveal the possible mechanism by which PP2C15 negatively regulates plant immunity, a Y2H screen was conducted between PP2C15 and known PTI signalling components, such as MAPK3/4/6, FLS2, NIK1, IOS1, BIK1, SERK1/2/4/5, and BAK1 (Figures 6; Figure S7). Among all combinations, only the kinase domain of BAK1 interacted with PP2C15, which was further confirmed by Co‐IP and LCA (Figure 6). An in vitro phosphatase assay showed that PP2C15 dephosphorylated BAK1‐KD cells (Figure 7).

**FIGURE 9:**
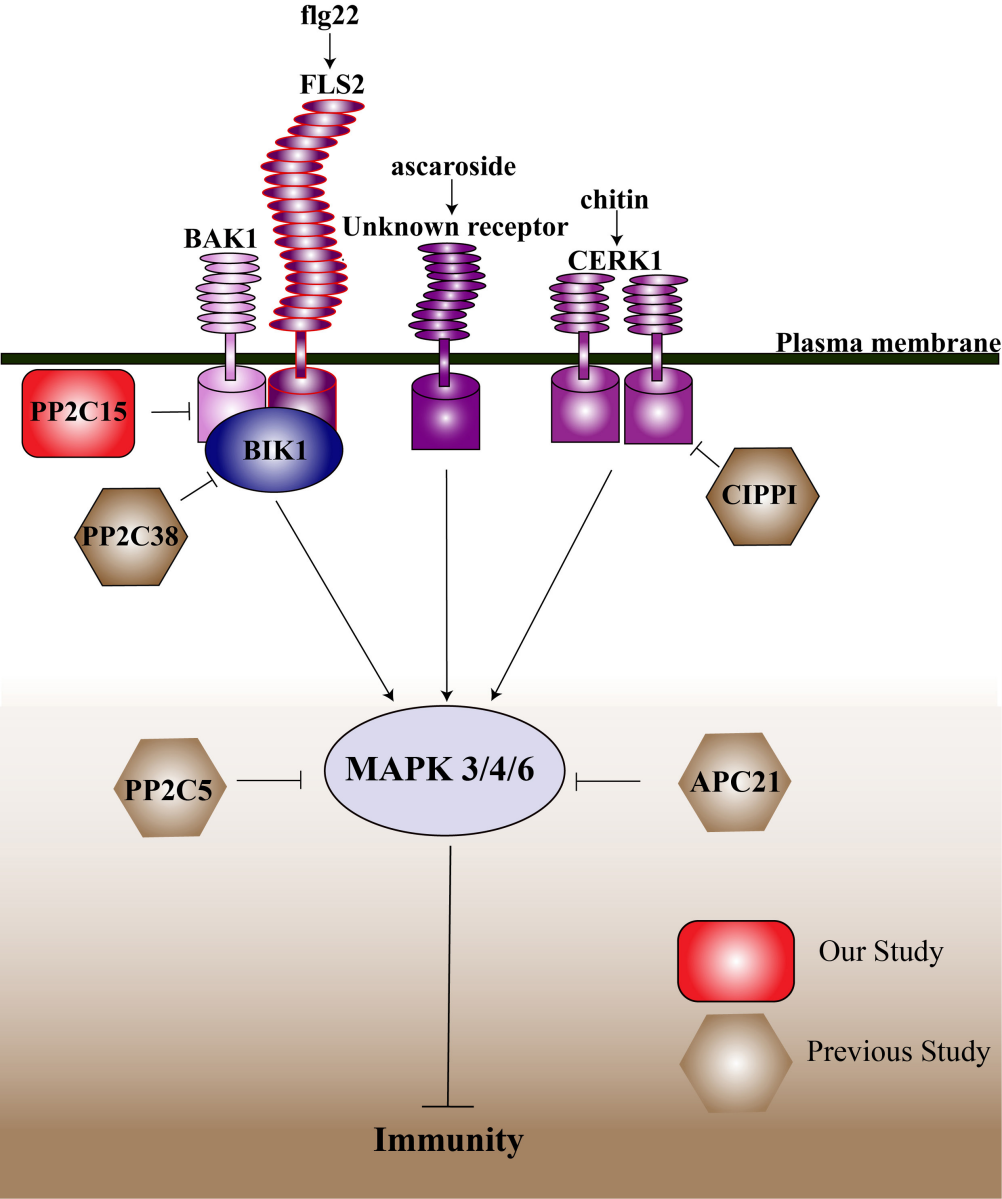
Schematic working model of PP2Cs on the negative regulation of the immune response.

Based on earlier reports and our findings, we summarize a simplified working model by which PP2Cs regulate *Arabidopsis* immunity (Figure 9). PAMP signals generated by bacteria, fungi, and nematodes, such as flg22, chitin, and ascaroside, are recognized by plasma membrane‐localized receptors, followed by the activation of co‐receptors and downstream signalling components (Bethke et al., 2012; Cheval et al., 2020; Denoux et al., 2008; Huang et al., 2020; Jelenska et al., 2017; Khokhani et al., 2021; Manosalva et al., 2015; Winkler et al., 2017; Yu et al., 2021). PP2C family members negatively regulate these pathways by targeting different components (Couto et al., 2016; Sidonskaya et al., 2016). AP2C1 participates in the negative regulation of nematode‐ and wound‐induced defence responses, although it is not clear whether AP2C1 functions downstream of ascaroside signalling (Ayatollahi et al., 2022; Sidonskaya et al., 2016). Currently, it is unknown whether PP2C5 plays a role in chitin‐or ascorbic glycoside‐induced PTI; however, our results indicate that it functions in flg22‐induced immunity and bacterial resistance by targeting MAPKs, which are core components of the PTI pathway. Contrastingly, PP2C38 and PP2C15 negatively regulate immunity by interfering with receptor complexes in the plasma membrane.

### 3.2 PP2C protein family members may positively regulate PTI

Cross‐talk exists in the regulatory networks of different signals, and proteins may play opposing roles in different biological processes. During screening, we noticed that three PP2C family members, SSPP, AT2G25070, and AT2G34740, upregulated the expression of the reporter by at least two‐fold (Figure 1a), indicating that these genes may positively regulate PT1. SSPP was first found to negatively regulate senescence via direct interaction and dephosphorylation of an LRR receptor‐like kinase, SENESCENCE‐ASSOCIATED RECEPTOR‐LIKE KINASE (AtSARK) (Xiao et al., 2015). However, studies found that overexpression of SSPP significantly improves salt tolerance and suppresses normal plant growth (You et al., 2022). Considering the convergence of biotic and abiotic stresses and the similar dwarfing phenotypes of plants with constitutive immunity, SSPP may act as a positive regulatory factor in immunity. However, this conclusion needs to be verified through functional studies (Ayatollahi et al., 2022; Cheng et al., 2011; Li et al., 1999, 2001; Shirano et al., 2002).

Previous studies have reported that transgenic tobacco plants overexpressing *OsBIPP2C1* and *OsBIPP2C2* show enhanced disease resistance against tobacco mosaic virus and *Phytophthora parasitica* (Hu et al., 2006, 2009). The overexpression of *ZmPP2C55* enhances drought stress tolerance in transgenic maize plants (Zhang et al., 2022). Therefore, PP2C protein family members may positively regulate plant responses, such as PTI, possibly by targeting negative regulators or signalling components of antagonistic pathways.

### 3.3 Negative regulators of plant immunity are potential targets for agricultural biotechnology

Globally, millions of tonnes of food crops are lost every year due to various diseases (Savary et al., 2019), and recent studies on the negative regulation of plant immunity suggest a possible way to achieve a higher degree of disease resistance. The RNA interference of potato StLecRK‐IV.1, which affects the stability of a positive immune regulator, reduces disease symptoms caused by *Phytophthora infestans* (Guo et al., 2022). OsTGAL1 interacts with the promoter of *OsSGT1*, which encodes a key enzyme in salicylic acid metabolism. *OsSGT1* knockout lines exhibit enhanced resistance to *Xanothomonas oryzae* (Li et al., 2022). A natural allele of a C2H2‐type transcription factor, *Bsr‐d1*, causes the binding of a repressive MYB transcription factor to its promoter, consequently reducing *BSR‐D1* expression and H_2_O_2_ degradation, and conferring non‐race‐specific resistance to blast in rice without reducing yield (Li et al., 2017).

PP2Cs represent more than 60% of the total phosphatase repertoire, with 80, 90, 91, and 88 genes identified in *Arabidopsis*, rice, tomato, and hot pepper genomes, respectively (Singh et al., 2016). Sequence and gene structure analyses of the PP2C family members in different plant species support their conservation in higher plants (Guo, Shi, et al., 2023; Wu et al., 2023). Similar to that observed in earlier studies (Ayatollahi et al., 2022; Couto et al., 2016; Sidonskaya et al., 2016), although mutant plants showed a more prominent response to immune signals, no autoimmunity was detected in *pp2c5* and *pp2c15* mutant plants (Figures 1, 2, 3, 4, 5). Although the growth and seed weight of *pp2c15* mutant plants were reduced, *pp2c5* mutants did not show any adverse effects compared with those of wild‐type plants. The CRISPR‐Cas9 gene‐editing technology (Gupta et al., 2019) was widely used in plant genome editing. This technology may be a valuable tool for plant breeders to knock out endogenous negative immune regulators such as PP2Cs to develop new cultivars with improved plant resistance without a yield penalty (Li et al., 2017).

In conclusion, our study revealed that PP2C15 negatively regulates PTI signalling by inhibiting BAK1 phosphorylation, thus affecting downstream signals, such as ROS production, callose deposition, stomatal closure, Pst DC3000 infection, and expression of defence‐related genes, ultimately leading to enhanced disease susceptibility in PP2C15 overexpression plants. In addition to PP2C15, many other PP2C genes, including PP2C5, inhibited *pFRK1*::*LUC* expression in *Arabidopsis* protoplasts. PP2C5 may act via the regulation of the MAPK cascade, although for the other PP2Cs it is unknown whether they interact with the signalling components of PTI, such as receptors or co‐receptors. With technological developments, we hope that the molecular mechanisms underlying the function of PP2Cs will be fully elucidated through future studies, thereby contributing to the breeding of crops with improved disease resistance.

## 4 EXPERIMENTAL MATERIALS AND METHODS

### 4.1 Plant materials and growth conditions

*Arabidopsis* plants used for ROS measurements, pathogen infection, stomatal closure experiments, and phenotype studies were planted in nutrient soil and grown at 24°C with 16 h of light for 4 weeks. For the callose and MAPK experiments, the seeds were sterilized with 75% ethanol and planted on 1/2× Murashige and Skoog (MS) plates. After 2 days of vernalization at 4°C, the seeds were cultured vertically for 10 days at 24°C and 16 h of light. The *Arabidopsis* plants used included Columbia wild‐type *Arabidopsis* (Col‐0), AT1G68410 overexpression lines (OE PP2C15), *pp2c15–1* mutant (*WiscDsLoxHs124_01C*) from NASC, *pp2c15–2* mutant produced by CRISPR/Cas9 (Figure S1), AT2G40180 overexpression lines (OE PP2C5), *pp2c5–1* mutants (Brock et al., 2010), and *pp2c5–2* mutants (SALK_015191). *N. benthamiana* plants used for the LCA and Co‐IP experiments were planted in nutrient soil and grown at 24°C with 16 h of light for 5 weeks.

### 4.2 Vector construction

Primers for constructing the 56 PP2C transient expression vectors, plant overexpression vectors, Y2H vectors, Co‐IP vectors, LCA vectors, and *pRSETA‐SUMO‐PP2C15* vectors were designed according to the instructions of the one‐step cloning kit (Vazyme). Table S1 summarizes the primer and vector information. *His‐MBP‐BAK1* ^*CD*^ and *His‐MBP‐BAK1* ^*CD+Km*^ (negative control) were prepared as previously described (Gong et al., 2019).

### 4.3 *Arabidopsis* protoplasts transient expression assay

Transient expression in *Arabidopsis* protoplasts was performed as described previously (He et al., 2006; Yoo et al., 2007). *HBT‐PP2Cs‐HAHA*, *pFRK1*::*LUC*, and *p35S*::*GUS* were transformed together into *Arabidopsis* protoplasts under the flg22 treatment and cultured for 12 h before detecting *pFRK1*::*LUC* and *p35S*::*GUS* activity. *HBT‐HAHA* was used as the control.

### 4.4 MAPK phosphorylation assay

The seeds of Col‐0, OE PP2C5 (OE‐1, OE‐2), OE PP2C15 (OE‐1, OE‐2), *pp2c5–1*, *pp2c5–2*, *pp2c15–1*, and *pp2c15–2* were grown vertically on 1/2× MS solid medium for 11 days after sterilization and vernalization. The cells were then transferred to a 1/2× MS broth containing 100 nM flg22 for 15 min. Seedlings were ground with protein extraction buffer (125 mM Tris–HCl, pH 6.8, 4% SDS, 20% glycerol, 5% β‐mercaptoethanol, 0.005% bromo‐formaldehyde) and incubated at 95°C for 10 min. Gel electrophoresis and immunoblotting were performed according to the standard protocols. MAPK phosphorylation was detected using a phospho‐p44/42 MAPK antibody, following the manufacturer's instructions.

### 4.5 RT‐qPCR

The *Arabidopsis* seedlings were cultured under the same conditions as those used for the MAPK assay. Seedlings grown vertically for 14 days were treated with 1 μM flg22 or water for 1 h. RNA was extracted according to the instructions of the M5 Quickspin universal plant RNA rapid extraction kit (Mei5bio). Reverse transcription was performed using the HiScript III RT Supermix for qPCR (+gDNA wiper) kit (Vazyme) according to the manufacturer's instructions. Experiments were performed using a Bio‐Rad Q5 qPCR. The primers used are listed in Table S1.

### 4.6 Subcellular localization

To visualize PP2C15 subcellular localization, the PP2C15‐GFP fusion protein was expressed in the leaves of 6‐week‐old *N. benthamiana* plants. Protein localization was visualized using a confocal laser scanning microscope (FV1200) as described by Majhi et al. (2019).

### 4.7 Callose deposition assay

*Arabidopsis* seedlings were cultured under the same conditions as those used for MAPK assays. The seedlings were treated with 100 nM flg22 for 20 h and rinsed with 95% ethanol overnight. On the second day, the seedlings were washed three times with 150 mM K_2_HPO_4_ and stained with a 50 mM K_2_HPO_4_ solution containing 0.01% aniline blue in the dark for 2 h. The fluorescence intensity was observed using a fluorescence microscope (BX51; Olympus).

### 4.8 Pathogen infection assay

Pst DC3000 cells were cultured in *Agrobacterium rhizogenes* broth (YEB) medium containing rifampicin and kanamycin at 28°C for 16 h. After centrifugation at 3000 *g* for 10 min, the cells were washed twice with sterile water. The cells were resuspended in 10 mM MgCl_2_. Pst DC3000 (10^5^ cfu/mL) was mixed with 300 nM flg22 and injected into 4‐week‐old *Arabidopsis* leaves using a 1‐mL needleless syringe. Leaves were collected on Days 0 and 3, and six rosette leaves per sample were punched (6 mm in diameter). After grinding in 200 μL 10 mM MgCl_2_, 10 μL spots were placed on Super Optimal broth (SOC) plates containing rifampicin and kanamycin. Colony numbers were counted after 2 days.

### 4.9 Stomatal opening assay

Four‐week‐old *Arabidopsis* leaves were incubated in stomatal buffer (20 mM KCl, 1 mM CaCl_2_, 2.5 mM MES‐KOH, pH 6.15) for 2–3 h in light (450 mol m^−2^ s^−1^). Water or flg22 with a final concentration of 4 μM was added to the samples. After 2 h of continuous illumination, the lower leaf epidermis was observed under a microscope.

### 4.10 DAB and Evans blue staining

Pst DC3000 (10^5^ cfu/mL) was injected into 4‐week‐old *A. thaliana* leaves. To detect ROS, the leaves were collected after 24 h and immersed in DAB solution (Vanacker et al., 2000). To detect cell death, leaves were collected after 72 h and immersed in Evans blue solution (Baker & Mock, 1994). The images were recorded using a Leica stereomicroscope. Relative cell activity was analysed according to a previously described method (Baker & Mock, 1994).

### 4.11 Y2H assay

Paired plasmids were transformed into the receptive states of *Saccharomyces cerevisiae* AH109 using the polyethylene glycol/lithium acetate method (Gietz & Schiestl, 2007). After being coated on SD − LW solid medium and cultured at 28°C for 3 days, single colonies were spotted on SD − LWH or SD − LWHA plates. X‐Gal staining was performed after 3 days of incubation at 28°C (Möckli & Auerbach, 2004).

### 4.12 Protein–protein interaction studies

Firefly LCA was performed as described (Zhou et al., 2018). For Co‐IP experiments, *PP2C15‐HAHA*, *BAK1‐GFP*, *GFP*, or the silencing suppressor *P19* (Voinnet et al., 2003) were transformed into *Agrobacterium tumefaciens* GV3101 that was cultured in YEB liquid medium containing rifampicin and kanamycin for 18 h at 28°C. The strains were resuspended in 10 mM MgCl_2_ and adjusted to OD_600_ = 1. Combinations of *PP2C15‐HAHA + P19*, *PP2C15‐HAHA + PP2C15‐GFP + P19*, and *PP2C15‐HAHA + BAK1‐GFP + P19* were mixed in equal proportions and added to 150 mM acetosyringone. Five‐week‐old leaves of *N. benthamiana* were injected with the above solutions using a needleless 1 mL syringe and cultured for 3 days. Thereafter, the leaves were ground to a powder with liquid nitrogen in the presence of a protein extraction buffer (Couto et al., 2016). Anti‐GFP magnetic beads (YaMei Biology) were added to the supernatant, and Co‐IP experiments were performed according to the manufacturer's instructions. Finally, the Co‐IP results were analysed by western blotting using anti‐GFP (GenScript) and anti‐HA (GenScript) antibodies.

### 4.13 Recombinant protein expression and purification

*Escherichia coli* BL21 cells (TransGen Biotech) containing prokaryotic expression constructs were grown at 37°C overnight in lysogeny broth (LB) medium and were transferred into 200 mL fresh medium at a ratio of 1:100. The bacteria were grown for 3–4 h at 37°C before the addition of 0.5 mM IPTG, and then cultured at 18°C for another 12 h for protein expression. 6 × His‐tagged recombinant proteins were purified using glutathione beads 4FF (Smart‐Lifesciences) or Ni‐NTA Sefinose (TM) resin (BBI) according to the manufacturer's instructions.

### 4.14 In vitro phosphatase assay

One microgram of purified recombinant His‐SUMO‐PP2C15 or His‐SUMO and 1 μg of purified recombinant His‐MBP‐BAK1^CD^ or His‐MBP‐BAK1^CD+Km^ (Gong et al., 2019) were mixed and incubated in a 25 μL reaction containing 250 mM imidazole (pH 7.2), 25 mM MgCl_2_, 1 mM EGTA, and 0.1% β‐mercaptoethanol for 4 h. Loading buffer was added to terminate the reaction. The phosphorylation status of His‐MBP‐BAK1^CD^ and His‐MBP‐BAK1^CD+Km^ was analysed using an anti‐pSer/Thr antibody (ECM Biosciences).

## CONFLICT OF INTEREST STATEMENT

The authors declare no conflict of interest.

## Supporting information


Figure S1.

Figure S2.

Figure S3.

Figure S4.

Figure S5.

Figure S6.

Figure S7.

Figure S8.

Table S1.

## Data Availability

Data supporting the findings of this study are available from the corresponding authors upon request.
