# Supplementary figures and images for: Functional screening of the Arabidopsis 2C protein phosphatases family identifies PP2C15 as a negative regulator of plant immunity by targeting BRI1‐associated receptor kinase 1
Source: Mol Plant Pathol. 2024 Apr 1;25(4):e13447. doi: 10.1111/mpp.13447 (PMC10984862; doi:10.1111/mpp.13447)

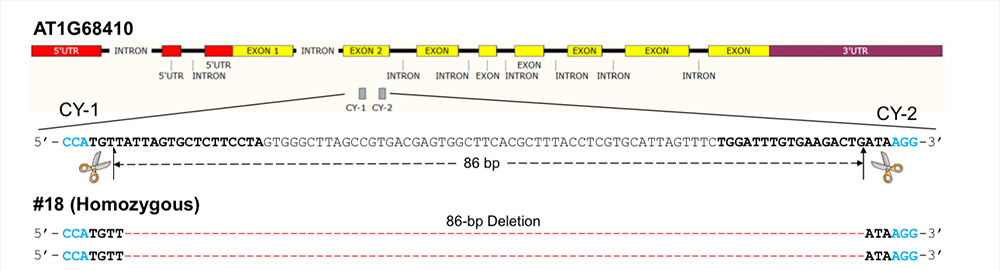

Supplement: Supplementary file 1 — Figure S1. [file MPP-25-e13447-s002.jpg]

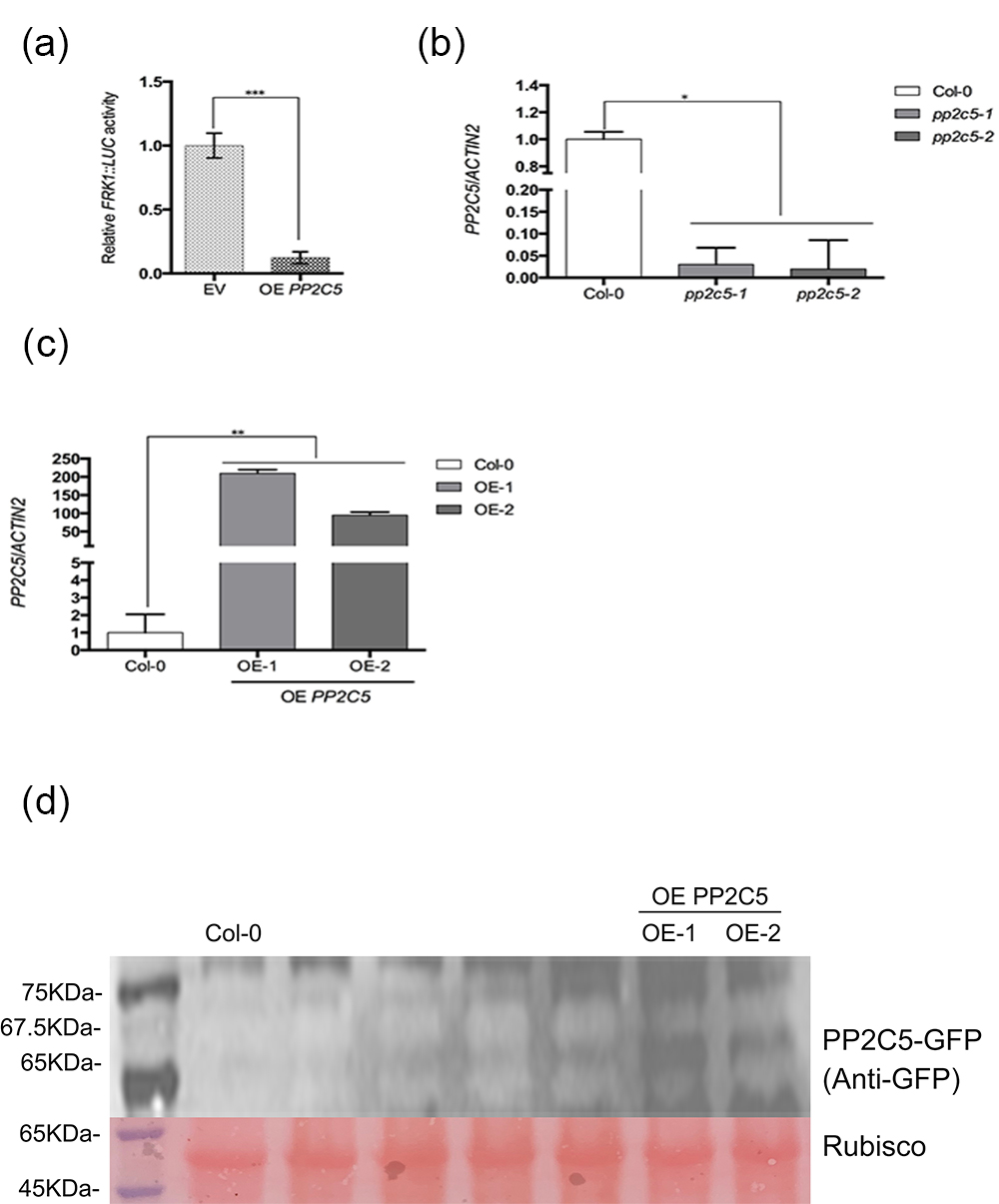

Supplement: Supplementary file 2 — Figure S2. [file MPP-25-e13447-s003.jpg]

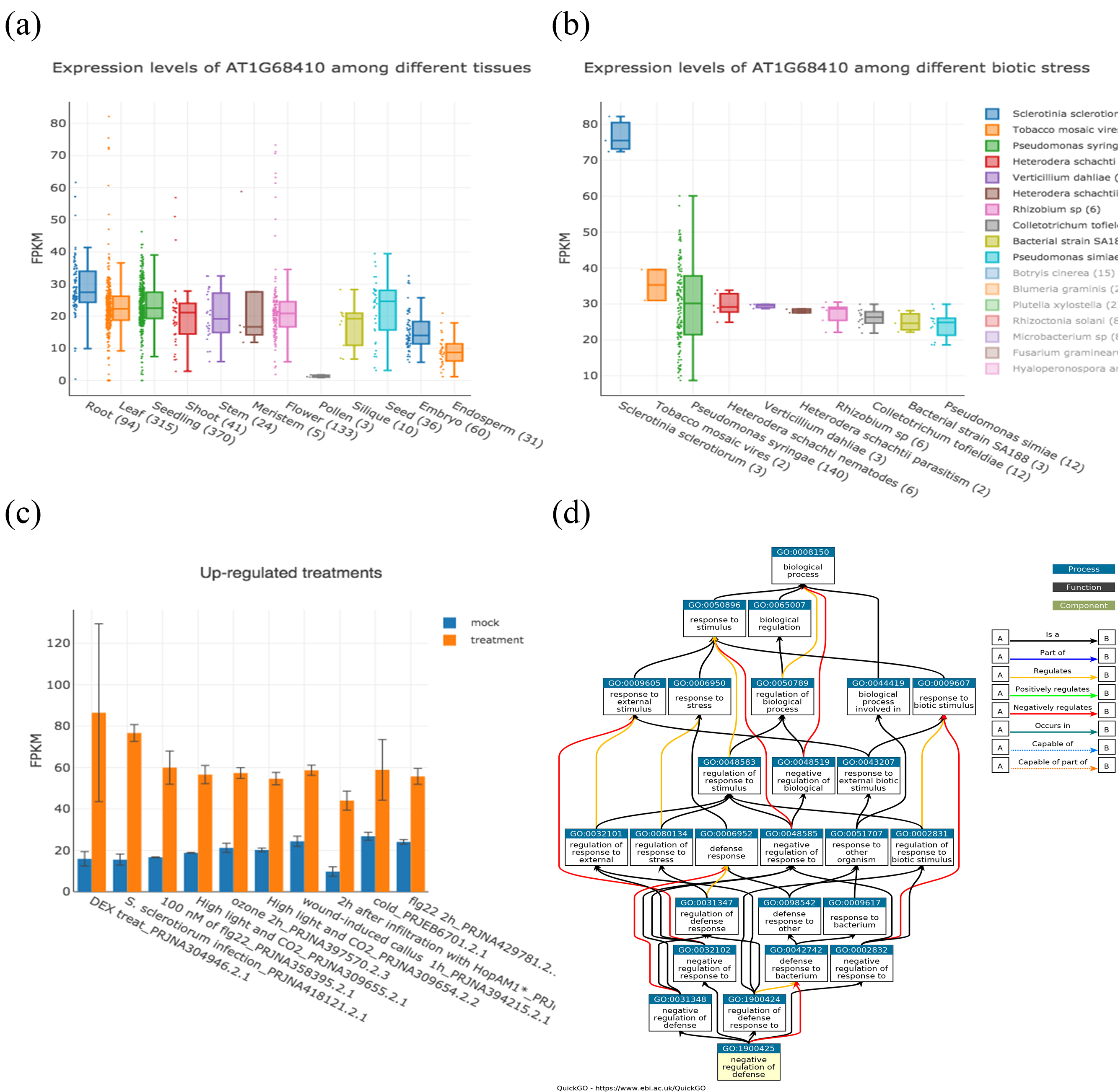

Supplement: Supplementary file 3 — Figure S3. [file MPP-25-e13447-s007.jpg]

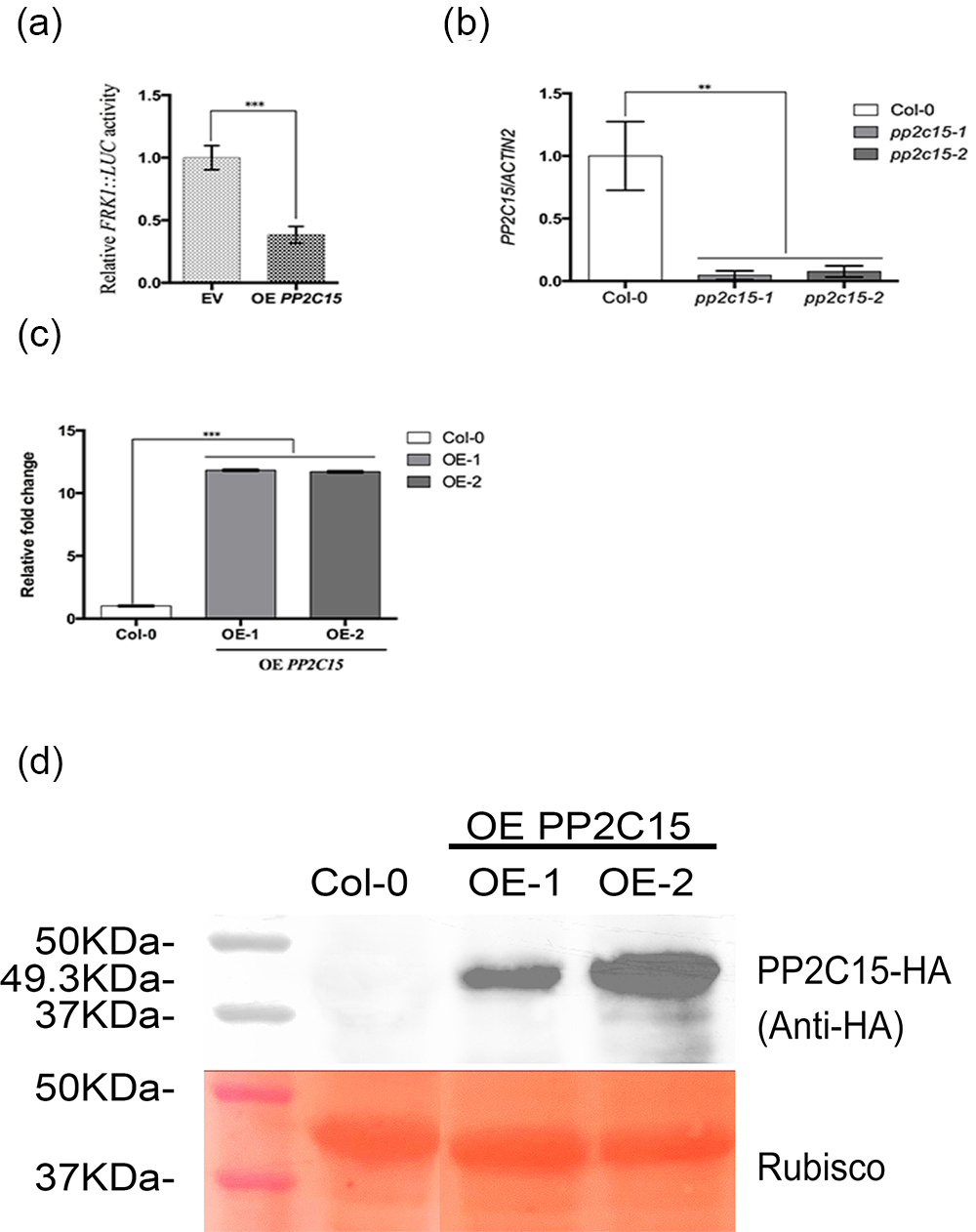

Supplement: Supplementary file 4 — Figure S4. [file MPP-25-e13447-s008.jpg]

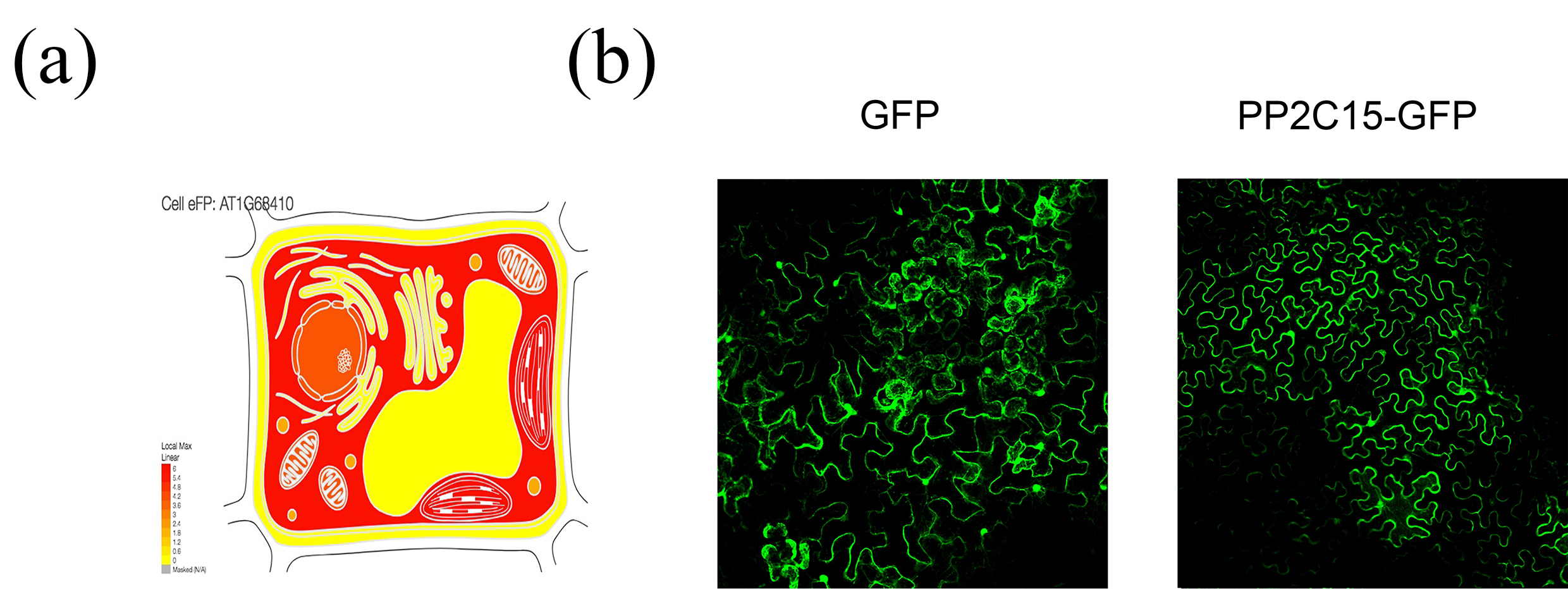

Supplement: Supplementary file 5 — Figure S5. [file MPP-25-e13447-s004.jpg]

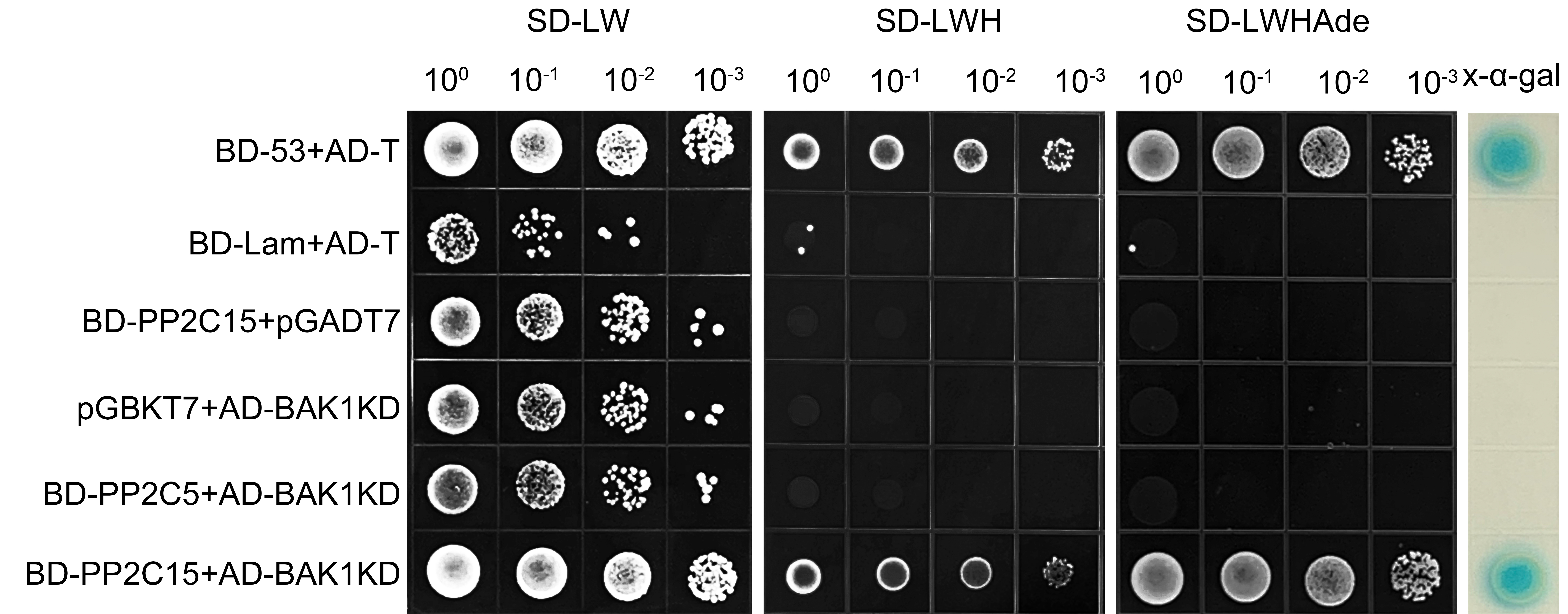

Supplement: Supplementary file 6 — Figure S6. [file MPP-25-e13447-s009.jpg]

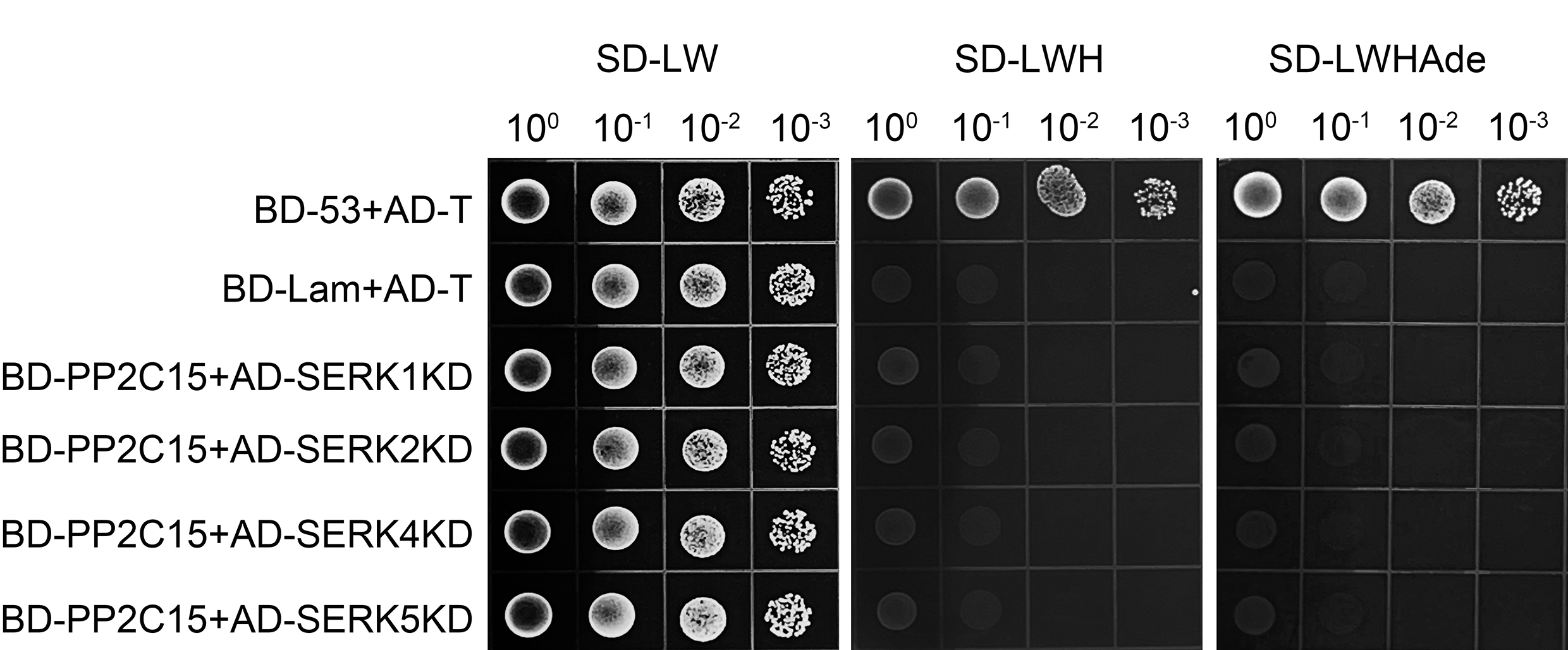

Supplement: Supplementary file 7 — Figure S7. [file MPP-25-e13447-s001.jpg]

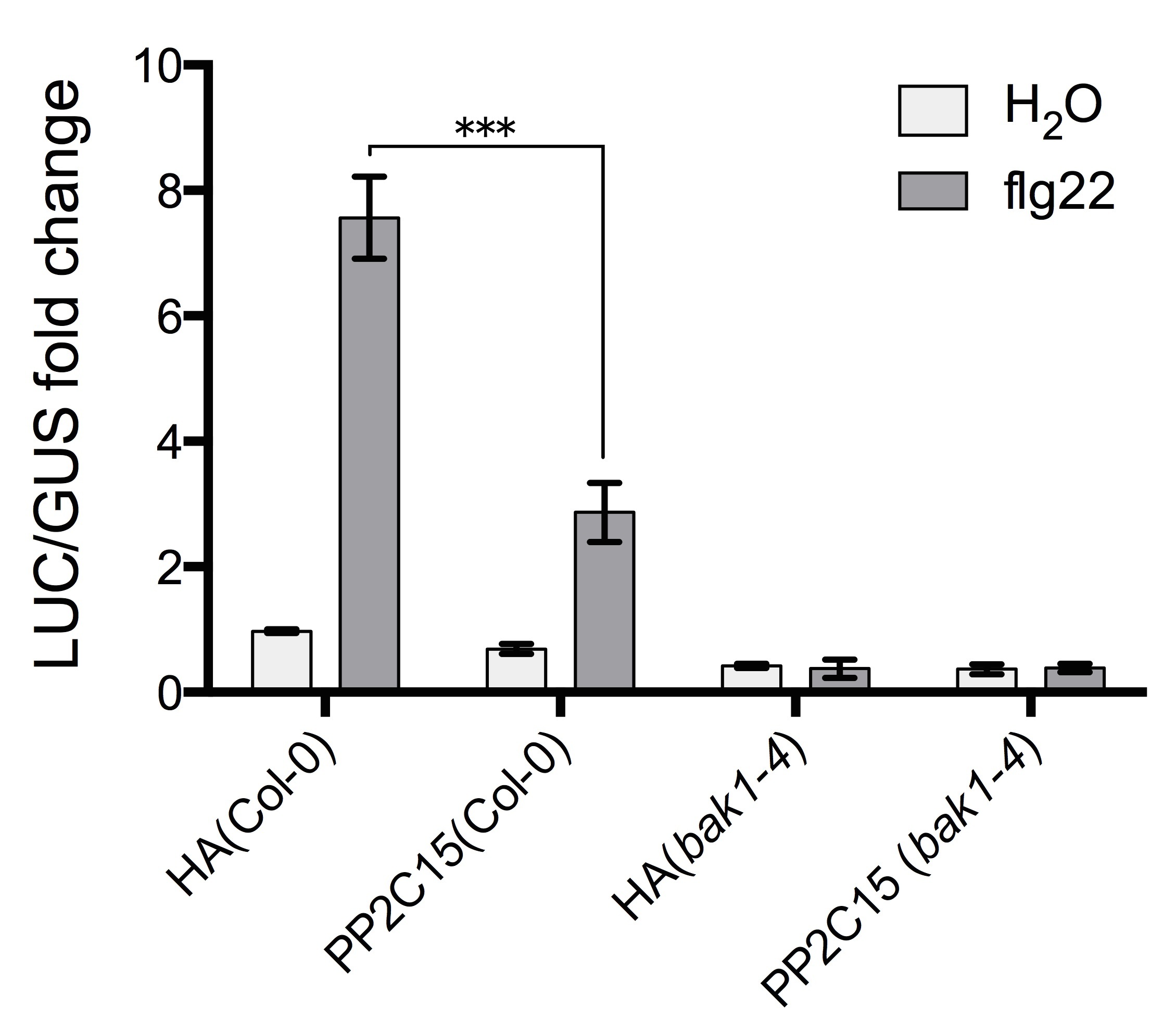

Supplement: Supplementary file 8 — Figure S8. [file MPP-25-e13447-s006.jpg]
